# SPANDx: a genomics pipeline for comparative analysis of large haploid whole genome re-sequencing datasets

**DOI:** 10.1186/1756-0500-7-618

**Published:** 2014-09-08

**Authors:** Derek S Sarovich, Erin P Price

**Affiliations:** Global and Tropical Health Division, Menzies School of Health Research, Charles Darwin University, PO Box 41096, Casuarina, 0811 NT Australia; Child Health Division, Menzies School of Health Research, Charles Darwin University, Casuarina, PO Box 41096, Darwin, 0810 NT Australia

**Keywords:** NGS, Haploid, Pipeline, Comparative genomics, Illumina, Ion PGM, Variant calling, SNP, Indel, Phylogeny

## Abstract

**Background:**

Next-generation sequencing (NGS) is now a commonplace tool for molecular characterisation of virtually any species of interest. Despite the ever-increasing use of NGS in laboratories worldwide, analysis of whole genome re-sequencing (WGS) datasets from start to finish remains nontrivial due to the fragmented nature of NGS software and the lack of experienced bioinformaticists in many research teams.

**Findings:**

We describe SPANDx (*S*ynergised *P*ipeline for *A*nalysis of *N*GS *D*ata in Linu*x*), a new tool for high-throughput comparative analysis of haploid WGS datasets comprising one through thousands of genomes. SPANDx consolidates several well-validated, open-source packages into a single tool, mitigating the need to learn and manipulate individual NGS programs. SPANDx incorporates BWA for alignment of raw NGS reads against a reference genome or pan-genome, followed by data filtering, variant calling and annotation using Picard, GATK, SAMtools and SnpEff. BEDTools has also been included for genetic locus presence/absence (P/A) determination to easily visualise the core and accessory genomes. Additional SPANDx features include construction of error-corrected single-nucleotide polymorphism (SNP) and insertion-deletion matrices, and P/A matrices, to enable user-friendly visualisation of genetic variants. The SNP matrices generated using VCFtools and GATK are directly importable into PAUP*, PHYLIP or RAxML for downstream phylogenetic analysis. SPANDx has been developed to handle NGS data from Illumina, Ion Personal Genome Machine (PGM) and 454 platforms, and we demonstrate that it has comparable performance across Illumina MiSeq/HiSeq2000 and Ion PGM data.

**Conclusion:**

SPANDx is an all-in-one tool for comprehensive haploid WGS analysis. SPANDx is open source and is freely available at: http://sourceforge.net/projects/spandx/.

## Background

The development of the first massively parallel next-generation sequencing (NGS) platform in 2005 [1] forever changed the medical and biological research landscape. A decade on, NGS technologies are now being routinely used for myriad purposes including whole-genome re-sequencing (WGS), genome-wide association studies, *de novo-* and re-assemblies, amplicon re-sequencing, polymorphism discovery, non-coding and coding RNA characterisation (RNA-seq), methylation studies (Methyl-seq) and protein-DNA interactions (ChIP-seq). The popularity of NGS has led to a rapid decrease in operating and reagent costs that have outstripped the “Moore’s law” paradigm, a common yardstick for measuring technological success based on computational hardware speed (http://www.genome.gov/sequencingcosts/). This plummeting cost has been brought about by major technological improvements and increased competition in the NGS platform market. Given continuing improvements in cost-effectiveness and versatility of NGS in molecular biology research, it is not surprising that NGS has become a mainstay in both small and large research laboratories across the globe.

The desire to answer important medical or biological questions using NGS, and in particular WGS, has concurrently driven the development of analysis tools designed to efficiently and accurately decode these vast volumes of nucleic acid data. However, analysis has been unable to keep pace with the volume of data being generated. Challenges to NGS data management and analysis include computation and storage availability and scalability, data sharing and privacy issues, NGS software costs and the requirement for bioinformaticists skilled in designing, programming and running complex analysis pipelines [2]. The technical difficulty and fragmented nature of NGS software, particularly for large-scale WGS analyses involving more than a handful of genomes, mean that comprehensive analyses remain out of reach for many researchers. In addition, the lack of transparent, publicly available and standardised NGS pipelines has potentially led to non-validated variant outputs being reported and perpetuated in the literature.

To address these issues, we have developed SPANDx (*S*ynergised *P*ipeline for *A*nalysis of *N*GS *D*ata in Linu*x*). SPANDx is an open-source, high-throughput, comparative genomic analysis tool for haploid organisms that integrates well-validated, open-source programs into a single program, thereby simplifying and standardising tedious WGS analysis workflows. SPANDx incorporates Burrows-Wheeler Aligner (BWA) [3, 4] for read mapping alignment, SAMtools [5] for read filtering and parsing, BEDTools [6] for genetic locus presence/absence (P/A) determination, Picard (http://picard.sourceforge.net) for data filtering, the Genome Analysis Tool Kit (GATK) [7, 8] for base quality score recalibration, variant determination, data filtering and improved insertion-deletion (indel) calling, VCFtools [9] for single-nucleotide polymorphism (SNP) and indel matrix construction, and SnpEff [10] for variant annotation. SPANDx has been written to analyse data generated from paired- and single-end Illumina (both pre- and post-v1.8 quality encoding) platforms, as well as Ion PGM and 454 single-end data.

SPANDx also incorporates several additional features aimed at minimising researcher hands-on time whilst enabling customisability. Most notably, SPANDx automatically generates a human-readable P/A matrix from individual BEDTools outputs, and can also construct error-corrected SNP and indel matrices when specified. These outputs enable quick and facile visualisation of genetic variants across a large number of genomes. SNP matrices generated by SPANDx are provided in .nex format and are directly importable into PAUP*, PHYLIP or RAxML for downstream phylogenetic analysis. Inbuilt, pre-optimised and customisable variant calling parameters for Illumina and Ion PGM data obviate the need for time-consuming optimisation of these settings, a requirement of other programs (e.g. Galaxy [11]). Unlike many WGS tools, SPANDx does not require the user to provide assembled genomes for every strain. SPANDx is run with a single command and parallelises many tasks by taking advantage of Portable Batch System (PBS) job scheduling, thereby reducing processing times for large datasets comprising tens through to thousands of genomes. Finally, SPANDx has been written in relatively simple, non-compiled, open-source code that enables users to customise the program by incorporating their preferred NGS tools (e.g. Bowtie [12] instead of BWA for read alignment), or by adding new features to its workflow.

## Findings

### SPANDx description

**Figure 1:**
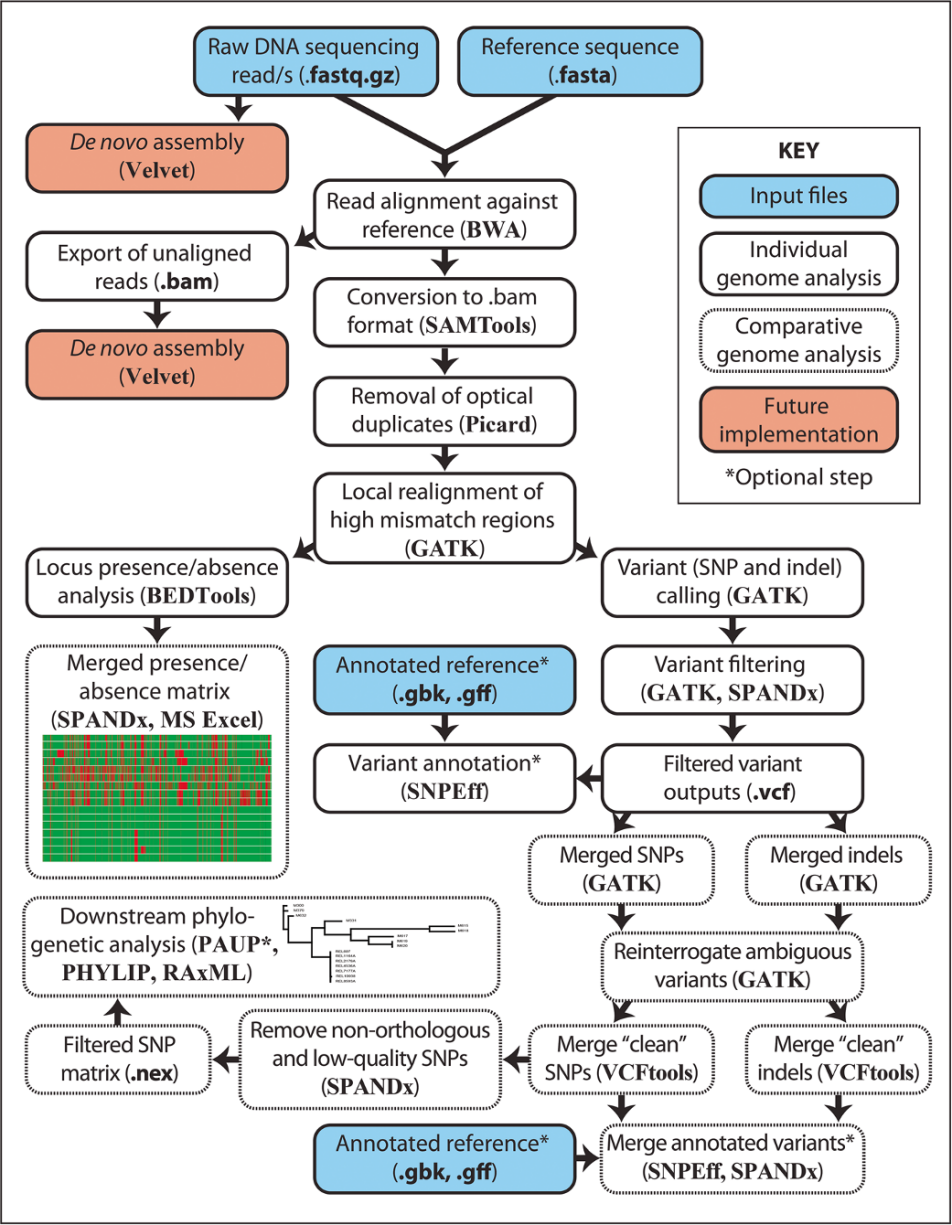
**SPANDx workflow for analysis of haploid next-generation re-sequencing data.**

The SPANDx workflow is shown in Figure 1. SPANDx is a shell package written for implementation in a Linux environment using Bash. SPANDx integrates multiple freely available Linux-based programs (BWA [3, 4], SAMTools [5], Picard, GATK [7, 8], VCFtools [9], BEDTools [6] and SnpEff [10]) into a single pipeline for alignment, variant identification, analysis and annotation from raw NGS data derived from haploid organisms. Using data generated from our prior WGS studies [13–16], we have tested the performance of SPANDx using paired-end Illumina (GA_*IIx*_, MiSeq and HiSeq2000) data, and single-end Ion PGM, Illumina, and 454 GS-FLX/FLX+ data. SPANDx is designed to run in a cluster environment and utilises parallel processing for the majority of the analysis pipeline. To facilitate parallelisation and appropriate resource allocation, SPANDx requires a Linux/UNIX system with PBS. The SPANDx user manual (available at: http://sourceforge.net/projects/spandx/) provides detailed information on installing, operating and where desired, customising this program.

### Variant identification and phylogenetic analysis

**Figure 2:**
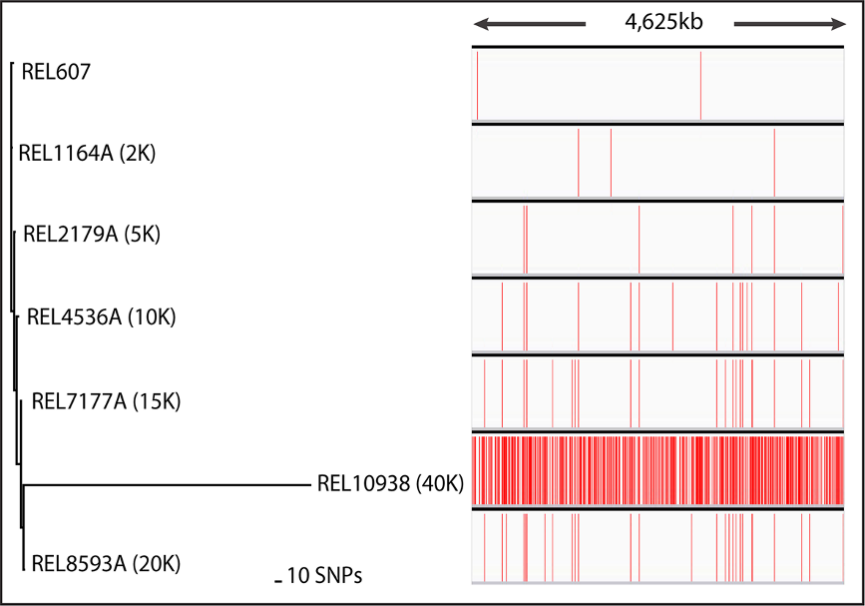
**Single-nucleotide polymorphism (SNP) variants identified by SPANDx across the genomes of seven clonal long-term** ***E. coli in vitro*** **passaged cultures.** The number of generations is indicated in parentheses. REL606, the ancestor for these passaged cultures, was used for reference genome comparison [GenBank:NC_012967]. As confirmed by SPANDx, REL607 is known to differ from REL606 by two SNPs [18], as denoted by the red vertical lines. In contrast, the ~40 K strain REL10938 is a hypermutable strain [19] and SPANDx identified 607 SNPs separating REL10938 from REL606. Phylogenetic analysis was performed using the Ortho_SNP_matrix.nex file, an output from SPANDx that can be directly imported into PAUP* 4.0 [17]. Using maximum parsimony, a highly accurate tree (consistency index = 1.0) was generated in PAUP*. SNPs were visualised with Integrative Genomics Viewer v2.3.25 [20].

**Figure 3:**
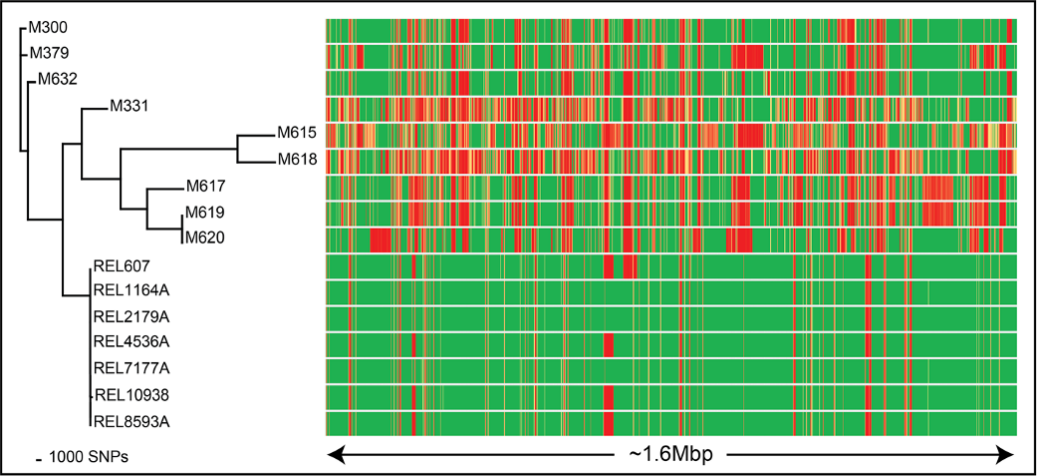
**Core single-nucleotide polymorphism (SNP) phylogenetic analysis across 16** ***E. coli*** **genomes (left), and comparison with the accessory genome (right).** The Ortho_SNP_matrix.nex file created by SPANDx was directly imported into PAUP* 4.0 and used for phylogenetic construction based on 106,557 core SNPs. Using maximum parsimony, a tree with a consistency index of 0.78 was generated. The Bedcov_merge.txt file for presence/absence analysis of loci was automatically generated by SPANDx using the coverageBED module of BEDTools [6], based on the default 1 kb window size. Regions with <95% coverage across one or more genomes are displayed, representing ~1.6Mbp of the *E. coli* genome (*x*-axis). Coverage is shown as a heat map, with red lines equating to low or no coverage through to green lines, which represent uniform coverage at each 1 kb window. In combination, these tools enable facile visualisation of the core and accessory haploid genomes.

**Figure 4:**
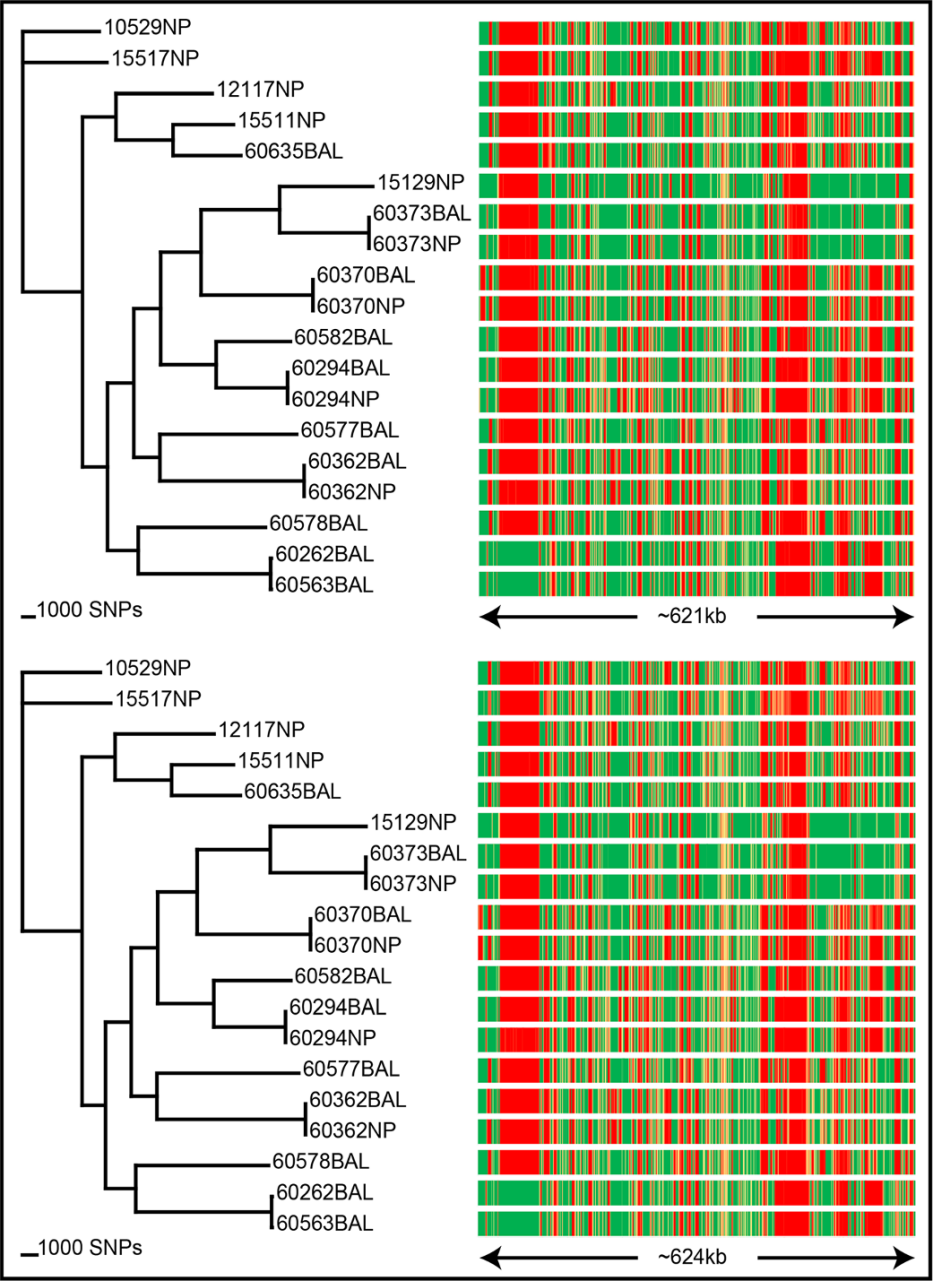
**Comparison of Illumina and Ion PGM platforms using SPANDx.** SPANDx was tested on 19 Australian *Haemophilus influenzae* strains [16] with both single-end Ion PGM and paired-end Illumina data. Strain 86-028NP [21] was used for reference alignment. From the Illumina data (top left), ~161,000 identified SNPs were used to construct a core genome SNP cladogram (CI = 0.47). From the Ion PGM data (bottom left), ~129,000 identified SNPs were used to construct a core genome SNP cladogram (CI = 0.48). The right-hand side panels show corresponding presence/absence data for each strain as described in Figure 3. For Illumina, 621 kb was found to be variable, compared with 624 kb with the Ion PGM data. Collectively, this comparison shows that SPANDx provides highly consistent haploid comparative genomic outputs across multiple NGS platforms.

Variant (i.e. SNP and indel) identification is a fundamental component of any haploid WGS analysis. For this study, default settings for SPANDx (as detailed in the user manual) were used to identify variants; optional settings were included as follows. The -m flag was used to construct core genome SNP matrices from the individual *Escherichia coli* or *Haemophilus influenzae* SNP .vcf files for phylogenetic reconstruction. The SPANDx-generated Ortho_SNP_matrix.nex file was directly imported into PAUP* 4.0b10 [17] and used to construct maximum parsimony phylogenetic trees (Figures 2, 3 and 4). For the seven REL *E. coli* genomes, SnpEff was implemented (using the -a and -v flags) to annotate SNPs.

### Presence/absence (P/A) analysis of *E. coli*and *H. influenzae*genomes

Defining the core (i.e. loci present in all taxa) vs. accessory (i.e. loci present in at least one taxon) genome is another fundamental application of haploid WGS. This information can be used for many purposes including pan-genome construction, strain-, species- or genus-level signature identification, or for observing patterns of genome reduction. The coverageBED module of BEDTools has been incorporated into the SPANDx pipeline for this purpose. BEDTools determines NGS read coverage depth and breadth across segments or ‘bins’ relative to the reference genome [6], thereby providing an efficient way of using raw NGS reads to identify both core and accessory genomic loci within a dataset compared with a reference genome. For the current study, a default 1 kb window size was used for P/A analysis. SPANDx automatically generates coverageBED genetic locus P/A outputs from all inputted genomes against the reference genome and combines individual outputs into a single human-readable matrix file (Bedcov_merge.txt). Additional file manipulation of P/A matrices was performed using basic features in MS Excel 2010 to create heat maps.

Example P/A matrices generated by SPANDx for the *E. coli* and *H. influenzae* datasets, which highlight the core and accessory genomes of these species compared with the reference genome, are respectively shown in Figures 3 and 4. We have previously used these outputs to develop a novel speciation target for *Burkholderia ubonensis* [13] and to characterise genome reduction in *Burkholderia pseudomallei* [14].

### Optimised variant calling for Illumina and Ion PGM data

Other NGS-based genomics tools such as Galaxy [11] require users to specify variant calling settings, which can be a subjective and time-consuming task, particularly for users unfamiliar with NGS data. To combat this issue, SPANDx includes pre-optimised variant calling for both single- and paired-end haploid NGS data across the Illumina and Ion PGM platforms. Although these settings have been optimised using our test datasets, they can be customised if desired by altering the filtering parameters in GATK.config, a file that comes with the SPANDx distribution.

### Phylogenetic analysis of SPANDx SNP outputs

Using a combination of VCFtools, GATK and several quality control and filtering steps, SPANDx automatically generates error-corrected core genome SNP matrices for phylogenetic analysis that can be directly imported into the phylogenetic programs PAUP*, PHYLIP and RAxML, the latter two of which are open-source software. The extensive error checking, filtering and variant identification steps undertaken in SPANDx using GATK ensure that the identified SNPs are as accurate as possible using NGS data. Example maximum parsimony analyses of SPANDx-generated data for the *E. coli* and *H. influenzae* datasets are shown in Figures 2, 3 and 4.

### Program comparison: SPANDx vs. BRESEQ

We performed an in-depth comparison of SPANDx with BRESEQ, a comparative genomics tool specifically designed for identifying SNPs, indels and large deletions in closely related microbial-sized genomes (http://barricklab.org/twiki/bin/view/Lab/ToolsBacterialGenomeResequencing). Due to limitations on BRESEQ data inputs, we only compared the six closely related *E. coli* REL genomes spanning 2 K to 40 K generations; REL607 was not included in the BRESEQ study [19] and was therefore excluded in this comparison. Default settings for SPANDx (as detailed in the SPANDx user manual) were used to identify variants. SnpEff was implemented using the -a and -v flags in SPANDx to annotate SNPs.

#### SNPs

SPANDx and BRESEQ identified identical SNPs for the 2 K, 5 K, 10 K, 15 K and 20 K mutants (3, 9, 16, 22 and 28 SNPs, respectively) [19]. One additional SNP in the 20 K strain, located at position 2129116 of *insB-15* in REL606, was not identified by either SPANDx or BRESEQ and was only discovered by Sanger sequencing [19]. This SNP was not able to be identified from NGS read data due to the paralogous nature of the *IS1* insertion sequence element in this genome. BLAST analysis of *IS1* in REL606 identified 27 highly related copies (>99% match across 100% of bases), with up to three SNPs present among the paralogues. Using NGS data, especially data harbouring relatively small insert sizes (~80-170 bp with this dataset), such loci cannot be accurately mapped. Therefore, the exclusion of this SNP from both the SPANDx and BRESEQ pipelines demonstrates the inherent limitations of using short-read NGS data for variant calling in large paralogous loci.

SPANDx analysis of the 40 K strain identified only 608 SNPs separating the hypermutable strain REL10938 from its REL606 ancestor, compared with the 626 SNPs found using BRESEQ. Closer examination found that these 18 SNPs were either not identified by SPANDx or were excluded using the default filtering parameters due to non-polymorphic (*n* = 1) or ambiguous (*n* = 11) genotypes, or poor mapping quality and/or insufficient (<0.5× of average) coverage (*n* = 6). The default parameters for SNP calling in SPANDx have been optimised such that the ability to identify only ‘real’ variants is maximised; false-positives are not tolerated with these settings, in line with GATK recommendations. Loosening of these parameters results in additional SNPs being identified, some of which may turn out to be ‘real’ upon confirmation with e.g. Sanger sequencing; however, the trade-off is that false-positives begin plaguing the dataset (results not shown). Given the nature of NGS data and the behaviour of NGS alignment programs, neither variant calling method is incorrect *per se*, but these minor differences between programs highlight the need to verify questionable SNPs from NGS data using secondary methods including manual inspection of NGS read alignments in e.g. Tablet [22], or wet laboratory-based analyses such as Sanger sequencing or allele-specific PCR.

#### Indels and chromosomal rearrangements

Comparison of SPANDx and BRESEQ for identifying small (<20 bp) indels in the REL strain cohort demonstrated that both methods were identical (variants are detailed in Supplementary Table two from [19]). Neither method identified a known 1.49Mbp inversion [23].

#### Large deletions and insertions

Large insertions are not currently able to be detected using SPANDx. However, for highly related strains these signatures can be detected with BRESEQ, as exemplified by the identification of ten IS element insertions with BRESEQ that were not found by SPANDx. Identification of large deletions (>20 bp) showed that, on a gross level, there was good consistency between the programs. However, the size of the deletions varied between SPANDx and BRESEQ, with SPANDx overestimating deletion size for three of the five identified deletions by ~0.7 to 1.4 kb. BLAST analysis of these regions showed that the additional sequence called as ‘deleted’ by SPANDx corresponded with paralogous IS element loci (results not shown). This finding was expected, being consistent with inherent read mapping difficulties across paralogous loci using short-read NGS data.

### Program comparison: SPANDx vs. Galaxy

Although we did not directly test Galaxy in this study, a previous study has used this program to compare *E. coli* strain REL607 with REL606 [18]. SPANDx identified that REL607 is a dual-nucleotide variant of REL606 at the *araA* and *recD* loci (Figure 2); no indels were found by either program. Thus, SPANDx confirmed previous variant findings identified using Galaxy [18].

### Cross-platform reproducibility of SPANDx

The performance of bioinformatics tools across multiple NGS platforms is an important consideration for analysis reproducibility and program utility. To address this question, we tested the performance of SPANDx using 19 *H. influenzae* strains subjected to two different NGS platforms: single-end Ion PGM and paired-end Illumina (MiSeq and HiSeq2000). SPANDx constructed almost identical core genome SNP phylogenies with these two datasets (Figure 4) despite being generated from platforms with inherently different error profiles and chemistries. In addition, P/A determination across these 19 genomes was essentially identical with these two platforms (Figure 4). These data demonstrate the robustness and accuracy of SPANDx across multiple NGS platforms.

## Discussion

SPANDx is a simple-to-use, high-throughput, and open source comparative genomics tool that has been developed for the integrated analysis of haploid WGS data from start to finish with minimal hands-on time. SPANDx has been written to handle multiple NGS platforms and currently can analyse single- and paired-end read data from the Illumina MiSeq/HiSeq/GA_*IIx*_ platforms, and single-end data from the Ion PGM and 454 GS FLX/FLX+ platforms. Because SPANDx uses PBS resource management, it has the capability of performing both single-core and parallel task processing, resulting in rapid turn-around-time, especially for medium- to large-scale WGS datasets comprising one, ten or even thousands of genomes.

SPANDx integrates existing, freely available comparative WGS analysis tools (BWA, Picard, the GATK, SAMTools, SnpEff, BEDTools and VCFtools) into a single pipeline. Importantly, SPANDx incorporates novel features for comprehensive analysis of raw haploid WGS data, and is aimed at simplifying downstream analysis (Figure 1) and increasing the user friendliness of data outputs. First, SPANDx automatically constructs P/A matrices of genetic loci using raw outputs generated by the coverageBED module of BEDTools. This feature enables identification of the core genome, a common goal of comparative haploid genome analyses. We have used this tool to design highly accurate species-specific assays for *B. ubonensis* [13], *H. influenzae* and *Haemophilus haemolyticus* (Price et al., manuscript in prep.), based on the identification of highly conserved loci that are absent in other species. Second, SPANDx can construct annotated, merged SNP and indel matrices from .vcf outputs. When a SNP matrix is generated, SPANDx will generate PAUP*, PHYLIP or RAxML-compatible outputs for downstream phylogenetic analysis (e.g. Figures 2, 3 and 4). Third, SPANDx contains pre-optimised yet customisable variant calling parameters for Illumina and Ion PGM data by default, allowing users to run analyses without spending a large amount of time optimising these parameters. These novel features of SPANDx enable users to quickly compare genomic data outputs without cumbersome and time-consuming manipulation of variant outputs.

Existing open-source comparative genomic tools for haploid NGS data analysis include Galaxy and BRESEQ. Galaxy (http://galaxyproject.org/) is a popular NGS tool that does not require any knowledge of Linux. The web-based version of Galaxy is particularly useful for small-scale analyses. Other advantages of Galaxy include its standardised outputs, frequent developer updates, cloud-based computer resource availability, and the ability to install the program locally where data privacy is of concern. The main limitation of Galaxy is the hands-on time required to construct an analysis pipeline, especially the need to manually optimise the filtering and data processing steps.

BRESEQ [19] is a command line tool implemented in C++ and R that is useful for finding variants (SNPs, indels, large deletions and new junctions supported by mosaic reads) relative to a closely related reference genome. Comparison of BRESEQ and SPANDx outputs in the current study demonstrated that both programs gave almost identical SNP and indel outputs, suggesting that both tools excel for this purpose. However, less consensus was found when identifying large deletion boundaries, with SPANDx overestimating deleted regions in 3/5 cases due to paralogous IS element loci flanking these regions, which cannot be mapped with short-read NGS data. BRESEQ has an additional advantage over SPANDx in its ability to identify larger (> ~20 bp) insertions, as SPANDx is not currently configured for this purpose. However, unlike SPANDx, BRESEQ is not appropriate for WGS analysis of more distantly related genomes or for medium- to large-scale datasets. Due to its lack of parallel processing, users of BRESEQ are limited to a reference genome of <20 Mb, an average genome coverage of <20×, and <1,000 expected mutations, and many comparative genomic functions are yet to be incorporated into its pipeline. BRESEQ also requires considerably more hands-on time to merge variant files than SPANDx and is thus not practical to use for more than a handful of genomes.

SPANDx has other advantages over existing tools and pipelines, including error-corrected SNP and indel matrices. To minimise effort and to standardise outputs across studies, SPANDx variant calling parameters have been optimised on our bacterial NGS datasets but can be customised to the user’s preference. Using default settings, we have demonstrated that SPANDx performs comparably for SNP calling across Illumina MiSeq/HiSeq2000- and Ion PGM-generated data. To the best of our knowledge, other pipelines have not been tested and validated across multiple NGS platforms.

Recognised shortcomings of SPANDx include the inability to identify SNP variation in paralogous regions, or inversions, although these issues were also identified in BRESEQ and are the result of NGS data and not an inherent shortcoming of these programs. Currently, SPANDx requires PBS to perform parallel processing and cannot be run on systems that do not possess this software. To increase the utility of SPANDx future versions will include the ability to run this pipeline with multiple resource handlers. Although SPANDx uses BEDTools for identifying large deletions, this program does not accurately pinpoint the exact positions of large deletions and further analysis is needed. SPANDx currently does not contain tools for identifying large insertions. For those wishing to identify chromosomal rearrangements or large (>20 bp) insertions, or to accurately characterise large deletions, it is recommended that genome assemblies are used instead of SPANDx (or similar programs).

## Conclusion

The NGS era has enabled researchers to generate unprecedented amounts of genomic data, but there remains a bottleneck in analysis. Genomic analysis pipelines such as SPANDx provide a streamlined way of decoding these data without the requirement for researchers to “reinvent the wheel” or learn multiple NGS programs. SPANDx is currently written to handle only haploid re-sequencing datasets. However, future development of SPANDx will include the ability to use other resource handlers (e.g. SGE), *de novo* assembly of accessory genome components from unaligned reads, *de novo* and reference-assisted genome assemblies, tools for insertion and chromosomal rearrangement detection and the ability to analyse diploid NGS data e.g. the human genome.

## Availability and requirements

**Project name:** SPANDx

**Project homepage:**https://sourceforge.net/projects/spandx/

**Operating system:** Linux

**Programming language:** Bash

**Other requirements:** Portable Batch System (TORQUE 2.5.13), Java 1.7.0_55, Burrows-Wheeler Aligner (BWA) 0.6.2, SAMtools 0.1.19, BEDTools 2.18.2, Picard 1.105, the Genome Analysis Tool Kit (GATK) 3.0 or higher, VCFtools 0.1.11, tabix 0.2.6 and SnpEff 3.6.

**License:** GNU General Public License version 3.0 (GPLv3)

**Any restrictions to use by non-academics:** Yes. Commercial users of GATK are required to obtain a licence for use. For further information, see http://www.appistry.com/gatk. As of version 3.1, GATK is open source to not-for-profit institutions only. SPANDx and all other software used by SPANDx are open source.

## Availability of supporting data

Two NGS datasets were used in this study. The first dataset comprised 16 publicly available *E. coli* Illumina HiSeq2000-generated genomes (Sequence Read Archive [SRA] accessions ERX287459, ERX287470, ERX287479, ERX287533, ERX287535 through ERX287538; ERX287540, SRX012986, and SRX012988 through SRX012993). Seven are isogenic ‘REL’ isolates from long-term evolution experiments (http://myxo.css.msu.edu/ecoli/) that span ~40,000 *in vitro* generations [19] and the additional nine are other more distantly related *E. coli* genomes from the SRA database. The FASTA file for the closed *E. coli* genome REL606 [19] was used as the reference for variant calling and annotation. The second dataset comprised 20 Australian *H. influenzae* strains sequenced using both the Ion PGM [16] and Illumina MiSeq [16] or HiSeq2000 platforms. The FASTA file for the closed *H. influenzae* 86-028NP genome [21] was used as the reference for variant calling.

## Abbreviations

NGS: Next-generation sequencing
WGS: Whole genome re-sequencing
SPANDx: Synergised Pipeline for Analysis of NGS data in Linux
SNP: Single-nucleotide polymorphism
BWA: Burrows wheeler aligner
GATK: Genome analysis tool kit
indel: Insertion-deletion
P/A: Presence/absence (of genetic loci).
